# Uncovering a Failed Pediatric Patient Population in Rural America: A Statewide Analysis of Over 1,000 Dog Bite Injuries

**DOI:** 10.7759/cureus.25734

**Published:** 2022-06-07

**Authors:** Sameer Massand, Marisa Giglio, Akshilkumar Patel, Chan Shen, Alexis Tashima, Elias Rizk, Thomas Samson

**Affiliations:** 1 Division of Plastic Surgery, Department of Surgery, Penn State Health Milton S. Hershey Medical Center, Hershey, USA; 2 College of Medicine, Pennsylvania State University, Hershey, USA; 3 Division of Outcomes Research and Quality, Department of Surgery, Penn State Health Milton S. Hershey Medical Center, Hershey, USA; 4 Division of Plastic and Reconstructive Surgery, Emory University School of Medicine, Atlanta, USA; 5 Department of Neurosurgery, Penn State Health Milton S. Hershey Medical Center, Hershey, USA

**Keywords:** rural, pediatrics, urban, animal bite, dog bites

## Abstract

Pediatric dog bites are prevalent and often devastating. Population-based data on these injuries can aid public health intervention efforts. However, most existing literature comes from single institutions in urban settings. We assess a statewide cohort to compare injury characteristics in urban and rural regions and find predictors for inter-hospital transfer. Data from 1,007 injuries from 2000 to 2015 were analyzed. Patients in rural areas were younger, more likely to be white and low-income, and more likely to receive delayed patient care. Injuries occurring in public settings as opposed to the private residence were more likely to involve males, occur in low-income areas, and involve non-white patients. Patients who required inter-hospital transfer were more likely to require a surgical subspecialist and operative repair. Our population analysis reveals children living in rural areas as a previously unidentified vulnerable patient population that may be suitable targets for public health interventions.

## Introduction

Dog bite injuries in the pediatric population are both prevalent and potentially morbid injuries [1]. For decades, surgeons and the broader health community have called for attention to these injuries. Some have gone as far as to advocate for a decrease in the number of dogs kept as pets in the United States, and others have suggested the singling out of breeds thought to be more violent [2-4]. Still, others have promoted the education of small children to better interact with domestic animals [5,6]. Regardless of these calls for action, dog bite injuries remain a significant public health concern.

Estimates as high as 4.5 million annual dog bite injuries in the United States have been cited, with one in five injuries requiring medical attention [7,8]. According to the American Society of Plastic Surgeons, over 26,000 dog bite repairs were performed in 2018 alone, making dog bite repair one of the 10 most frequently performed reconstructive procedures overall. In emergency departments across the United States, it is invariably among the 20 most frequent chief complaints [9]. Pediatric patients are disproportionately affected, with half of all dog bite injuries occurring in children [10]. There is a 50% likelihood of sustaining a dog bite during childhood and a 20% likelihood of sustaining a dog bite injury in adulthood [7,11]. These injuries account for 1.5% of all visits to U.S. emergency departments [12].

While most dog bite injuries result in minor injuries requiring only local wound care, some can be severe and lead to long-term functional, aesthetic, and psychosocial consequences, as well as, on rare occasions, death [13]. As such, abundant research has been performed to identify potential targets for intervention. Many have found that younger male children are most frequently injured and that the offending canines are most encountered at home and are known to the patient. Existing literature, however, has generally described single institution experiences reporting primarily from urban populations [10,14]. Those that examine larger datasets do so without distinguishing injuries in rural areas from those in higher population areas [15,16]. In this study, we broaden the sample population to include the entire state of Pennsylvania, allowing us to examine differences in injuries occurring in patients from urban versus rural areas with attention to patient demographics and location, as well as the timing of the injury. Additionally, we set out to assess if any factors predict the need for the transfer of these patients to referral facilities as a means to expedite their future care.

## Materials and methods

A retrospective review of children with dog bite injuries was performed using data collected from the Pennsylvania Trauma Systems Foundation from 2000 to 2015. This database requires data entry and reporting for any patients treated for a diagnosis of trauma at trauma centers of any designation within Pennsylvania. Dog bite injuries were identified from this database by searching for records using the International Classification of Disease (ICD) system, using the ICD-9 code E906.0 and ICD-10 code W54.0, both of which represent dog bite injuries. The records were then evaluated for the age of the patient, and only pediatric patients, aged zero to 18 years, were included in the study. All patients older than 18 years or children who were bitten by animals other than dogs were excluded. Data from 1,007 pediatric dog bite injuries were obtained. For each case, the following information was collected: patient demographics (including age, sex, race, and patient zip code), the county in which the injury occurred, place the attack occurred (private residence versus public), time of day in which the injury occurred, and the time elapsed between dog bite injury and hospital arrival. We categorized the time of day into two categories: day (i.e., between 5 am and 9 pm) or night (i.e., after 9 pm before 5 am). Private residence included any home, regardless of the relation of the patient to that home.

Using the 2013 Rural-Urban Continuum codes provided by the United States Department of Agriculture (USDA), a designation of urban or rural was given to each dog bite injury based on the patient’s home county. Counties with rural-urban continuum codes between one and three were given a designation of urban, and codes between four and nine were given a designation of rural. This correlated with the USDA designation of “metro” and “non-metro.” Additionally, using the U.S. Census Bureau’s 2017 American Community Survey, the per capita income in the past 12 months was determined based on patient zip code. Per capita income for each record was then sorted into three categories: low-income (i.e., less than $30,000), high-income (i.e., greater than $30,000), or unknown.

Further variables identified from the dataset were Injury Severity Scale, interhospital transfer, procedure location, and procedure service. The Injury Severity Scale was previously assigned at the time of entry to the database and is based on abbreviated injury scale scores to identify the overall severity of the event. For this study, this was further broken down to define “regular” and “more severe” as a score of one and anything greater than one, respectively. The Interhospital transfer was marked affirmatively if the dataset included any vital signs or arrival time data from two institutions. Procedure location was separated into two categories - one for a formal operating room setting and the other for all other locations such as the emergency department, procedure room, or at the bedside in any hospital unit. Lastly, the service performing the procedure was identified in each recorded injury that required repair. For this study, all surgical subspecialties (i.e., plastic surgery, otolaryngology, oral and maxillofacial surgery, ophthalmology) were grouped into one category and analyzed as such.

Statistical analysis

We provide sample frequencies and percentages, and we used t-test and chi-square tests to examine subgroup differences by urban or rural status. A multivariable logistic regression excluding patients with unknown elapsed time was used to examine the probability of having a longer elapsed time (greater than 120 minutes versus less than 120 minutes). Similarly, a multivariable logistic regression excluding patients with the unknown location of injury was used to examine the probability of injury location being public. To examine the use of interhospital transfers, we characterized the sample by whether a transfer occurred and provided sample frequencies, percentages, and t-test and chi-square test results. Further, we employed a multivariable logistic regression to identify factors associated with the use of interhospital transfers. All statistical analyses were conducted in SAS 9.4 (SAS Institute Inc, Cary, NC, USA).

## Results

Urban vs. rural

We provide patients’ demographic characteristics by urban or rural status in Table 1. Out of the 1,007 injuries in our study sample, the majority (n = 828, 82.2%) occurred in patients living in urban areas, while 180 (17.8%) occurred in those living in rural areas. Subgroup analysis reveals that cases involving patients living in rural areas were more likely to involve younger patients (6.2 years vs. 7.3 years, p = 0.001), patients who are white (88.3% vs. 68.8%, p < 0.001), and patients from a low-income area (57.8% vs. 49.4%, p < 0.001). Lastly, more patients in this group experienced a longer elapsed time to medical care (62.8% vs. 36.4%, p < 0.001). Injuries in the rural subset trended toward being more severe (46.7% vs. 38.8%, p = 0.05) and occurring in a private residence more frequently (87.2% vs. 79.5%, p = 0.054), although these differences were not significant. There was no difference in sex among patients in the two settings (58.3% male vs. 56.5% male, p = 0.656) or the time of day the injury occurred (91.7% day vs. 93% day, p = 0.533).

**Table 1:** Demographic characteristics of urban vs. rural patients. SD: Standard Deviation

	Urban (N=828)	Rural (N=180)	p-value
Age			0.001
Mean (SD)	7.3 (4.64)	6.2 (4.56)	
Race			<0.001
White	570 (68.8%)	159 (88.3%)	
Non-White	181 (21.9%)	10 (5.6%)	
Unknown	77 (9.3%)	11 (6.1%)	
Sex			0.656
Male	468 (56.5%)	105 (58.3%)	
Female	360 (43.5%)	75 (41.7%)	
Injury Type			0.050
Regular	507 (61.2%)	96 (53.3%)	
More Severe	321 (38.8%)	84 (46.7%)	
Injury Location			0.054
Private Residence	658 (79.5%)	157 (87.2%)	
Public	82 (9.9%)	12 (6.7%)	
Unknown	88 (10.6%)	11 (6.1%)	
Income			<0.001
Low	409 (49.4%)	104 (57.8%)	
High	419 (50.6%)	0 (0%)	
Unknown	0 (0%)	76 (42.2%)	
Time of Injury			0.533
Day	770 (93%)	165 (91.7%)	
Night	58 (7%)	15 (8.3%)	
Time Elapsed to Medical Care			<0.001
<120 min	221 (26.7%)	26 (14.4%)	
120+ min	301 (36.4%)	113 (62.8%)	
Unknown	306 (37%)	41 (22.8%)	

Table 2 provides the logistic regression results for the longest elapsed time. The results confirm that patients living in rural areas still had a higher likelihood of having a longer elapsed time to medical care (odds ratio, OR = 1.92, p = 0.021) after controlling for other covariates. The pseudo R2 value for this logistic regression was 0.08. Chi-square analysis also demonstrated significant relationships between several independent variables, including race and income (p = 0.01), race and location (p < 0.001), and location and income (p < 0.001).

**Table 2:** Logistic regression results for longer elapsed time (120+ minutes) to medical care. OR: Odds Ratio

Variable	OR	95% Confidence Interval	
Race: White vs. Other	2.329	1.497	3.624
Sex: Male vs. Female	1.270	0.908	1.777
Rural vs. Urban	1.915	1.047	3.503
Income: Low vs. High	0.957	0.667	1.373

Public vs. private residence

Table 3 provides results from the logistic regression for the probability of injury location being public (versus private residence). The sample size for this analysis was 908, as we excluded cases with unknown location information. It showed that females (versus males, OR = 0.41, p = 0.001) and high-income areas (versus low-income, OR = 0.24, p = 0.014) were less likely to have injury location being public. Non-white race (versus white, OR = 3.59, P = 0.003) was significantly associated with a higher likelihood of dog bites in public. The pseudo R2 value for this logistic regression was 0.08.

**Table 3:** Logistic regression results for the probability of injury location being public (versus private residence). OR: Odds Ratio

Variable	OR	95% Confidence Interval	
Race: Other vs. White	3.592	2.171	5.943
Sex: Female vs. Male	0.407	0.245	0.676
Urban vs. Rural	2.336	0.789	6.916
Income: High vs. Unknown	0.237	0.060	0.942
Income: Low vs. Unknown	0.430	0.120	1.540

Interhospital transfer

Patients’ demographic characteristics by interhospital transfer are listed in Table 4. Patients who required hospital transfer were more likely to undergo repair in the operating room (50.1% vs. 24.4%, p < 0.001) and by a surgical specialty (52.6% vs. 25.3%, p < 0.001). Table 5 shows the results from logistic regression for the probability of interhospital transfer. It confirms that after controlling for covariates, the interhospital transfer is still significantly associated with the case being evaluated by a surgical subspecialty versus general surgery (OR = 3.37, p < 0.001); interhospital transfer cases were also less likely to undergo repair at bedside versus a formal operating room (OR = 0.50, p < 0.001). The urban or rural status was not significantly associated with interhospital transfer (p = 0.754). The pseudo R2 value for this logistic regression was 0.10.

**Table 4:** Demographic characteristics of patients by interhospital transfer.

	Interhospital transfer		
	Yes (N=561)	No (N=446)	p-value
Sex			0.515
Male	226 (53.6%)	265 (59.4%)	
Female	196 (46.4%)	181 (40.6%)	
Urban:Rural Status			0.934
Urban	345 (81.8%)	365 (81.8%)	
Rural	77 (18.2%)	81 (18.2%)	
Time of Injury			0.873
Day	391 (92.7%)	413 (92.6%)	
Night	31 (7.3%)	33 (7.4%)	
Injury Location			0.226
Home	278 (49.6%)	224 (50.2%)	
Other’s home	179 (31.9%)	133 (29.8%)	
Public	44 (7.8%)	50 (11.2%)	
Unknown	60 (10.7%)	39 (8.7%)	
Income			0.454
Low	295 (52.6%)	217 (48.7%)	
High	226 (40.3%)	193 (43.4%)	
Unknown	40 (7.1%)	36 (8.1%)	
Time Elapsed to Medical Care			0.28
<120 min	132 (25.5%)	115 (25.8%)	
120+ min	243 (43.3%)	171 (38.3%)	
Unknown	186 (33.2%)	160 (35.8%)	
Procedure Location			< .001>
Bedside	280 (49.9%)	337 (75.6%)	
Operating Room	281 (50.1%)	109 (24.4%)	
Procedure Service			< .001>
Trauma/General surgery	28 (5%)	46 (10.3%)	
Surgical subspecialties	295 (52.6%)	113 (25.3%)	
Non-surgical subspecialty	104 (18.5%)	135 (30.3%)	
No surgery	134 (23.9%)	152 (34.1%)	
Race			0.784
White	402 (71.7%)	326 (73.1%)	
Non-White	107 (19.1%)	84 (18.8%)	
Unknown	52 (9.3%)	36 (8.1%)	
Injury Type			0.089
Regular	247 (58.5%)	262 (58.7%)	
More Severe	175 (41.5%)	184 (41.3%)	

**Table 5:** Logistic regression results for the probability of interhospital transfer. OR: Odds Ratio

Variable	OR	95% Confidence Interval		p-value
Urban: Rural Status				
Urban vs. Rural	1.080	0.669	1.743	0.752
Procedure Location				
Bedside vs. Operating Room	0.497	0.343	0.719	<0.001
Procedure Service				
Surgical Subspecialty vs. Trauma/General Surgery	3.368	1.954	5.805	<0.001

## Discussion

Dog bite injuries remain a public health concern as they are not only frequent but also devastating, with both pediatric and adult victims experiencing severe morbidity and mortality [1,3,13,17]. Reports estimate that 4.5 million dog bite injuries occur in the United States annually; although this number is declining, the international incidence rate continues to rise, indicating a growing global health burden that physicians must often help manage [18,19].

As a result, broad efforts have been proposed to mitigate these injuries. Although many programs have aimed preventative, education-based interventions at children, such as teaching proper behavior around dogs, many have assumed a one-size-fits-all approach [5,6,20,21]. While our study corroborates many existing findings, it indicates heterogeneity in the demographic and clinical characteristics of dog bite injuries and questions the suitability of such generic approaches.

The literature indicates male children are most at risk, a finding that aligns with our results for both urban and rural regions [14]. Regarding age, some studies have shown that children less than five years old are most at risk, while others have pointed to grade school children, ages six to 12 [22-24]. Our results indicate these disparate results may be attributable to regional differences; children in urban areas are, on average, 1.1 years older than children in rural areas (average age of 7.3 versus 6.2 years). One possible explanation for this is the trend toward a higher frequency of public injuries (p = 0.054), which are likely to involve older children [25]. It should be noted that regardless of frequency, injuries in the youngest cohorts tend to be more severe and centered on the head and neck region [26,27].

Researchers have generally agreed that patients are more likely to be bitten by dogs familiar to them [25,26]. If we extrapolate that injuries occurring in a private residence are inflicted by familiar dogs, this finding is affirmed by our results. Interestingly, the difference may be even starker in rural areas, where 87.2% of injuries occurred in a private residence, as opposed to 79.5% in urban areas, although this difference was not statistically significant (p = 0.053). This is further reflected in the finding that non-white patients are more frequently bitten in public in urban settings.

Racial disparities in dog bite injuries have been examined and reported less frequently than other demographic variables [10,16,26]. Those that do report race demographics have cited a white patient component of between 60% and 75% [9,15]. We present this data not only as a composite but also as it breaks down into urban and rural populations. Overall, the finding of 72.3% of cases involving white, non-Hispanic patients in our study, which loosely reflects the overall 76.1% white population in Pennsylvania, is consistent with the existing data [28]. Examining this granularly with attention to urban and rural areas, however, provides a more nuanced picture. The incidence of dog bite injuries in white children was found to be higher in the former compared to the latter. Additionally, rural areas have a preponderance of low-income patients as proxied by zip code median income. These two findings can likely be explained by demographic differences in the broader populations of urban and rural areas. One previous Canadian study has indicated that dog bites are more frequent in low-income areas even when controlling for urbanity [29].

Examining injuries inflicted upon patients from rural versus urban areas reveal that patients in rural areas have a significantly longer time for evaluation (62.8%) than patients in urban areas (36.4%). To determine if this disparity was compounded by the need for transfer to referral institutions, which may not be as readily available in rural areas, we investigated the predictors of the need for transfer. Interestingly, geography did not have any effect on the need for transfer (p = 0.754), while injuries requiring evaluation by a subspecialty and necessitating a procedure in the operating room did influence the need for transfer. This finding indicates that only injuries requiring formal operations by subspecialists are transferred to referral facilities, where the necessary operating room staff and subspecialists may be more readily available. It is quite possibly a combination of both factors.

The inter-hospital transfer has many effects on care to consider. Patients who require transfer are inherently delayed in their time to treatment. Costs are incurred, and resources are consumed by both the patient and the healthcare system. One-fourth of transfers included a distance traveled of greater than 50 miles [30]. Ultimately, however, its function is to best match specialty care with a patient’s condition. By identifying early those patients who are likely to require a transfer, changes in practice may allow for the optimization of these variables, potentially mitigating the ramifications on time and resources. Because neither injury severity, location of event, age, gender, income group, nor time of day were found to be significant predictors of need for transfer, providers must instead continue to rely on an assessment of whether or not an injury will require specialty evaluation and formal operation as a gauge for the need for transfer.

This study has some limitations. It is a retrospective analysis using an established patient database, and as such, gaps are inherent in both data collection and reporting. Given the population of the state and the known frequency of dog bite injuries, our cohort of just over 1,000 injuries is likely incomplete. This may represent a skew toward more severe injuries, which are more likely to necessitate medical care, though we can only speculate. Additionally, using the zip code of patients’ primary residence to estimate their socioeconomic status is imprecise as the population in a zip code is not perfectly homogenous. The determinations on socioeconomic status were made based on publicly available data by zip code and are not patient-specific, which dilutes the clinical conclusions that we can make. We are also limited by the data in conducting a thorough subgroup analysis to further contextualize patients’ income level and dog bite injury risk as multiple additional confounders, such as more rural patients being white, may be present. Although zip codes are often used in the literature as a corollary of household income, further investigation into the granular characteristics of rural versus urban zip codes would supplement the findings of this study.

## Conclusions

Our study of dog bite injuries reveals a subpopulation of pediatric patients in rural areas that have otherwise not been recognized as distinct. Patients who suffer dog bite injuries in these areas are younger, more frequently white, low-income, and further from medical attention. These demographic disparities are likely to be present across the United States and other developed nations where a similar rural-urban continuum exists, potentially confounding efforts for limiting the number and severity of injuries. Other nations should perform their own population analyses to identify the exact demographic differences that may manifest between rural and urban populations so that interventions can be applied appropriately. These interventions can be tailored to serve the distinct populations that exist within the rural-urban continuum. Such interventions might take the form of advocacy targeting dog owners, delivering school-based education to young children, providing dog training classes free of charge, and many others. Dog bite injuries are common in all developed nations and are deserving of intervention. All physicians caring for pediatric patients should be aware of the demographic and clinical characteristics of the patients they are likely to treat.
